# Kolmogorov complexity metrics in assessing L2 proficiency: An information-theoretic approach

**DOI:** 10.3389/fpsyg.2022.1024147

**Published:** 2022-10-06

**Authors:** Gui Wang, Hui Wang, Li Wang

**Affiliations:** Foreign Languages College, Shanghai Normal University, Shanghai, China

**Keywords:** linguistic complexity, L2 writing, learner corpus, language assessment, syntactic complexity, morphological complexity, language proficiency, information theory

## Abstract

Based on 774 argumentative writings produced by Chinese English as a foreign language (EFL) learners, this study examined the extent to which Kolmogorov complexity metrics can distinguish the proficiency levels of beginner, lower-intermediate, and upper-intermediate second language (L2) English learners. Kolmogorov complexity metric is a holistic information-theoretic approach, which measures three facets of linguistic complexity, i.e., overall, syntactic, and morphological complexity simultaneously. To assess its validity in distinguishing L2 proficiency, Kolmogorov complexity metric is compared with traditional syntactic and morphological complexity metrics as well as fine-grained syntactic complexity metrics. Results showed that Kolmogorov overall and syntactic complexity could significantly distinguish any adjacent pair of L2 levels, serving as the best separators explored in the present study. Neither Kolmogorov morphological complexity nor other complexity metrics at both the syntactic and morphological levels can distinguish between all pairs of adjacent levels. Results of correlation analysis showed that Kolmogorov syntactic complexity was not or weakly correlated with all the fine-grained syntactic complexity metrics, indicating that they may address distinct linguistic features and can complement each other to better predict different proficiency levels.

## Introduction

Recent years have seen a rapid growth of quantitative studies in assessing learners’ writing performance. Despite the multifaceted nature of second language (L2) writing performance, researchers have examined it primarily from three aspects: complexity, accuracy, and fluency (CAF) (Wolfe-Quintero et al., 1998; Ellis, 2003; Ortega, 2003; Norris and Ortega, 2009).

Among these three aspects, linguistic complexity has been extensively explored in the field of second language assessment. A considerable amount of literature has delved into the relationships between linguistic complexity and L2 proficiency or L2 writing quality (Lu, 2011; Kyle and Crossley, 2017, 2018; Brezina and Pallotti, 2019; Khushik and Huhta, 2019; Bulté and Roothooft, 2020; Ouyang et al., 2022; Zhang and Lu, 2022). In this line of research, a primary concern lies in identifying valid and reliable complexity metrics that can effectively predict different L2 learners’ proficiency levels or developmental stages (Egbert, 2017; Kyle and Crossley, 2017; Lu, 2017).

Previous studies have revealed various powerful complexity metrics in different dimensions. Specifically, at the level of syntactic complexity, length-based metrics (e.g., mean length of sentences, clauses, and T-units) have been demonstrated to perform well when pinpointing different proficiency levels (Wolfe-Quintero et al., 1998; Ortega, 2003; Lu, 2011, 2017). More recent studies have addressed the need for fine-grained syntactic complexity metrics and assessed their abilities in predicting the quality of written or spoken production (Kyle and Crossley, 2017, 2018). Concerning lexical metrics, lexical diversity metrics such as Guiraud’s index, type-token ratio (Kettunen, 2014; De Clercq, 2015; Treffers-Daller et al., 2016; Bulté and Roothooft, 2020) and newly proposed lexical sophistication metrics such as n-gram association strength (Kim et al., 2017; Kyle et al., 2017) have proved to be effective in capturing differences associated with L2 proficiency.

To summarize, although these studies are informative and significant, they have centered principally on syntactic or lexical complexity. Little attention has been paid to which and to what extent morphological complexity metrics can be a reliable index of proficiency (Brezina and Pallotti, 2019). In addition, although the fine-grained complexity metrics have been proved to be valid and reliable in assessing L2 writing quality (usually measured by scores), their effectiveness in distinguishing more global L2 proficiency levels (e.g., beginner, intermediate, and advanced) is still open to debate.

To this end, we propose to use a holistic information-theoretic approach named Kolmogorov complexity, which is defined as the length of the shortest possible description required to regenerate the running texts and can be approximated by using file compression programs (Ehret, 2021). More importantly, Kolmogorov complexity is able to address the three sublevels of linguistic complexity at the same time (i.e., overall, syntactic, and morphological complexity), thus serving as an effective complement to the above-mentioned fine-grained complexity metrics.

Therefore, the present study aims to examine the validity of Kolmogorov complexity in indexing L2 learners’ proficiency by comparing it with the traditional syntactic and morphological complexity metrics as well as fine-grained syntactic complexity metrics at overall, syntactic, and morphological complexity levels. Furthermore, we have investigated the correlation among complexity metrics at different levels, which helps us understand the reason why some metrics are able to distinguish L2 proficiency and which metrics should be selected for better assessing learners’ proficiency levels.

## Literature review

### The relationship between linguistic complexity and L2 proficiency

Linguistic complexity has been extensively examined as an index of linguistic performance, development, and proficiency in L2 learners. Nevertheless, no consensus regarding the definition of complexity has been reached, given that the word “complexity” has been assigned to various closely related meanings in second language acquisition (SLA) research (Bulté and Housen, 2012, 2014; Ortega, 2012; Pallotti, 2015).

Bulté and Housen (2012) put forward an elaborate taxonomy to reveal the multifaceted nature of L2 complexity. Drawing from the theoretical discussions of complexity (e.g., Dahl, 2004; Miestamo et al., 2008), Bulté and Housen (2012) divided L2 complexity into relative and absolute complexity. Relative complexity is a subjective and agent-related notion that refers to the cognitive cost (or difficulty) of processing experienced by a language user (Miestamo et al., 2008), while absolute complexity is objective and defined by the formal properties inborn in a linguistic system (Bulté and Housen, 2012). To further elaborate learners’ L2 performance in SLA research, Bulté and Housen (2012) distinguished the broader notion of absolute complexity into propositional complexity, discourse-interactional complexity, and linguistic complexity. What we focus on in the present study is the linguistic complexity that denotes an absolute, objective, and essentially quantitative property of language units, features, and (sub)systems (Bulté and Roothooft, 2020). More precisely, we adopted the Kolmogorov complexity, which is defined as the length of the shortest description that can reproduce the sample text (Li et al., 2004; Juola, 2008).

A major strand of research in linguistic complexity of second language writing has centered on the relationship between linguistic complexity and L2 proficiency. Relevant studies have yielded rich and insightful findings about which and to what extent various complexity metrics correlate with or reliably index L2 proficiency.

To be specific, at the syntactic level, numerous metrics have provided mixed results in capturing differences related to learners’ language proficiency (e.g., Wolfe-Quintero et al., 1998; Lu, 2010; Graesser et al., 2011; Biber et al., 2014; Khushik and Huhta, 2019; Barrot and Agdeppa, 2021). Much earlier L2 syntactic complexity research has examined the usefulness of holistic length-based (e.g., mean length of sentences, clauses, and T-units) or clausal-level (e.g., dependent clauses per clause) syntactic complexity metrics for indexing L2 proficiency or development (e.g., Wolfe-Quintero et al., 1998; Ortega, 2003). However, due to the lack of computational tools to automate complexity analysis, the research mentioned above is limited to a small scale of syntactic complexity metrics and learner samples.

To promote larger-scale data analysis, Lu (2010) developed the L2 Syntactic Complexity Analyzer (L2SCA) to automate the analysis using 14 traditional metrics covering four dimensions, namely, length of production unit, subordination, coordination, and degree of phrasal complexity. A growing number of studies have investigated the predictive power of these metrics and claimed that length-based metrics fared better when pinpointing the different L2 proficiency levels (Lu, 2011, 2012; Chen et al., 2013; Li, 2015; Yang et al., 2015; Ouyang et al., 2022).

Despite the fact that several metrics were found to correlate significantly with proficiency levels, recent research has highlighted some concerns regarding their use in assessing L2 writing performance. Firstly, it has been argued that traditional syntactic complexity metrics failed to capture the emergence of particular structures in language development (Larsen-Freeman, 2009; Taguchi et al., 2013; Biber et al., 2014; Kyle and Crossley, 2018). Secondly, many syntactic metrics hitherto applied in assessing L2 writing proficiency might be more prototypical of spoken than written language (Biber et al., 2011; Kyle and Crossley, 2018). Biber et al. (2011) proposed that informal conversations were characterized by clausal complexity, whereas academic writing was characterized by phrasal complexity.

Thus, to further address the above-mentioned issues, recent studies have shifted their attention to more fine-grained features that take the structural types of clauses and phrases into consideration and have demonstrated their predictive power over traditional syntactic complexity metrics (e.g., Biber et al., 2014; Kyle, 2016; Kyle and Crossley, 2017, 2018).

For instance, Kyle (2016) developed the Tool for the Automatic Analysis of Syntactic Sophistication and Complexity (TAASSC), which comprises 31 fine-grained metrics of clausal complexity, 132 metrics of phrasal complexity, and 190 usage-based metrics of syntactic sophistication. Subsequently, Kyle and Crossley (2017, 2018) explored the reliability of fine-grained metrics in indexing English as a foreign language (EFL) writing quality scores by comparing their metrics with traditional syntactic complexity metrics from the L2SCA.

In both studies, the mean length of clause (MLC) explained 5.8% of the variance in holistic essay scores, serving as the only traditional metric incorporated in regression models. Concerning fine-grained metrics, Kyle and Crossley (2017) found that four syntactic sophistication measures (e.g., average lemma construction frequency and average delta P) fitted into the regression model, accounting for 14.2% of the variance in holistic essay scores. Kyle and Crossley (2018) reported that one fine-grained clausal complexity metric (i.e., nominal subjects per clause) and six fine-grained phrasal complexity metrics (e.g., prepositions per object of the preposition and adjectival modifiers per object of the preposition) fitted into the regression model, together interpreting 20.3% of the variance. These results demonstrated the merits of using fine-grained metrics, especially those associated with phrasal complexity, in predicting L2 writing quality, which is consistent with Biber et al. (2011). In addition, Zhang and Lu (2022) further supported the findings of Kyle and Crossley (2017, 2018) by taking genre differences into consideration. Specifically, Zhang and Lu (2022) found that fine-grained metrics accounted for a larger variance in writing quality scores than traditional metrics for both application letters and argumentative essays.

Lexical complexity is usually operationalized through three dimensions: lexical density, lexical diversity, and lexical sophistication. Among these metrics, lexical density was poor at capturing variations related to proficiency levels, as proposed by previous studies (e.g., Green, 2012; Lu, 2012; Crossley and McNamara, 2013; Park, 2013). By contrast, lexical diversity, a metric of how many distinct words are used in the text, stands out as a reliable indicator of proficiency. For instance, Treffers-Daller et al. (2016) found that the number of different words, the type-token ratio (TTR), and the Guiraud’s index displayed strong associations with proficiency levels. By examining the development of lexical complexity at four proficiency levels, De Clercq (2015) reported that D and Guiraud’s index served as the best separators for different L2 proficiency. More recent studies have concluded that lexical sophistication metrics such as n-gram association strength increased throughout language development (Kim et al., 2017; Kyle et al., 2017).

Compared with previous research on syntactic and lexical metrics, fewer studies have examined L2 learners’ production at the morphological level. For instance, based on the written argumentative essays collected from the Louvain Corpus of Native English Essays (LOCNESS) and the International Corpus of Learner English (ICLE), Brezina and Pallotti (2019) examined how Morphological Complexity Index (MCI; Pallotti, 2015) varied across English native speaker and advanced English learners. Surprisingly, no statistically significant difference was found on the MCI between native speakers and L2 learners. De Clercq and Housen (2019) investigated how three morphological complexity metrics, namely, Malvern et al.’s (2004) Inflectional Diversity (ID), Pallotti’s (2015) Morphological Complexity Index (MCI), and Horst and Collins’ (2006) Type/Family Ratio (T/F), changed in oral L2 French and L2 English across four proficiency levels. Their results found that those measures could only discriminate between L2 English learners at low proficiency levels. Whereas these studies were certainly informative, the metrics involved only depict one aspect of morphology (i.e., inflectional complexity), leaving derivational complexity underexplored.

In short, the studies reviewed have provided a rich and varied picture of the relationship between various linguistic complexity metrics and L2 proficiency or production quality. However, most of these studies have fixed their eyes on either lexical or syntactic complexity, with limited empirical studies targeting morphological complexity metrics.

In this respect, we propose to use Kolmogorov complexity, a holistic and operationally convenient approach, which can deal with the three sublevels of linguistic complexity (i.e., overall, morphological and syntactic complexity) simultaneously. In addition, we expect that Kolmogorov complexity may complement the above-mentioned complexity metrics and provide some insightful findings to current literature.

### Kolmogorov complexity

Kolmogorov complexity is a concept originating from information theory that deals with defining and quantifying information (Der, 1997). Shannon (1948), who proposed the first quantitative measurement of information, stated that information could be determined according to its uncertainty, or more specifically, the entropy involved in selecting a message from a variety of choices. Founded on the notion of entropy, Kolmogorov complexity is put forward to analyze the information content of a string of words or symbols instead of a range of alternative messages.

The Kolmogorov complexity of a text can be assessed by the length of the shortest description to restate it (Li et al., 2004; Juola, 2008). Although several mathematical issues prevent the direct calculation of Kolmogorov complexity (Kolmogorov, 1968), the entropy estimation approach can be used to approximate its computation. Such an approach can be realized by file compression programs like gzip, whose algorithms are based on the structural redundancies and regularities of the running texts/strings. To be specific, the first step of compression in gzip is to back-reference the redundant string along with the length of the duplicated string and the distance from its previous occurrence (Ziv and Lempel, 1977). Then, using the statistical compression method *Huffman coding*, these length-distance pairs and the unique strings are further compressed (Salomon, 2004). Simply put, this program first “loads” a given number of texts and then “stores” them in a temporary lexicon. As the program further processes the texts, previously occurred sequences will be recognized based on the temporary lexicon and then be compressed to remove redundancies (Ehret, 2014).

Linguistically speaking, Kolmogorov complexity is inconsistent with traditional linguistic complexity metrics. The latter focuses on particular structures and grammatical features, and the occurrence of certain grammatical patterns, such as dependent clauses and relative clauses, is always indicative of complex writings (Biber and Gray, 2016). In contrast, Kolmogorov complexity is not feature-based but global and holistic since it considers the entire structural complexity of sample texts. In other words, this complexity metric is not related to deep linguistic form-function pairings but to structural surface redundancy or the recurrence of orthographic character sequences within a text (Ehret, 2021: 387–388).

Kolmogorov complexity was initially employed by the mathematician Juola (1998) and later applied in linguistic research. At an early stage, Kolmogorov complexity was adopted to investigate cross-linguistic complexity variations by analyzing parallel corpora that contain the original sample texts as well as their translations (Juola, 2008; Sadeniemi et al., 2008; Ehret and Szmrecsanyi, 2016).

In addition, Kolmogorov complexity was also demonstrated to be applicable to non-parallel corpus data, thus providing a methodological basis for the present study. Ehret (2021), for example, found that Kolmogorov complexity could be a useful indicator of register formality by examining both written and spoken registers of British English in the British National Corpus. It is noteworthy that formal registers are characterized by a higher level of overall and morphological complexity but a lower level of syntactic complexity than informal registers. In addition, Ehret and Szmrecsanyi (2019) employed Kolmogorov complexity to analyze naturalistic second language acquisition data. Results showed that Kolmogorov overall and morphological complexity scores increased with the amount of instruction received by L2 learners.

Additionally, other information-theoretical metrics have been used to examine L2 complexity. Sun and Wang (2021) employed the relative entropy of linguistic complexity to examine the development of L2 learner proficiency. They concluded that relative entropy was a better measure of proficiency than traditional algorithms based on frequency summation or ratio. It should be noted, however, that Sun and Wang (2021) focused only on lexical and grammatical aspects, with minimal attention paid to morphological complexity. Paquot (2017) used the scores of Pointwise Mutual Information (PMI) to measure collocational complexity of phraseology, which is overlooked in previous L2 complexity research. However, PMI can only be used to measure associations between two groups of events; it cannot be used to capture other kinds of linguistic phenomena (Sun and Wang, 2021).

Against these backdrops, the present study seeks to contribute to the existing research concerning the reliability of complexity metrics in differentiating L2 proficiency levels. Specifically, we adopted an information-theoretic Kolmogorov complexity approach and examined its validity in indexing L2 proficiency by comparing it with traditional syntactic and morphological complexity metrics, and fine-grained syntactic complexity metrics. The research questions are as follows:

(1) To what extent can Kolmogorov complexity metrics (i.e., overall, syntactic, and morphological complexity) differentiate L2 learners’ proficiency levels, as compared with traditional syntactic and morphological as well as fine-grained syntactic complexity metrics?

(2) Are there any correlations between all these complexity metrics at different levels?

## Methodology

### Corpus data

To determine the effects of complexity metrics at various levels in predicting L2 proficiency, we used the International Corpus Network of Asian Learners of English (ICNALE; Ishikawa, 2011, 2013)-Written as our corpus. The reason for choosing this corpus is threefold.

Firstly, the ICNALE-Written, comprising 5,600 written essays and amounting to 1.3 million tokens, is the largest international learner corpus focusing on Asian learners’ English. In addition, this corpus is produced by 2,600 beginner to advanced learners from ten Asian countries/regions, who are divided into four Common European Framework of Reference (CEFR)-linked proficiency levels: A2_0 (beginner), B1_1 (lower-intermediate), B1_2 (upper-intermediate), and B2+ (advanced). These proficiency levels were determined by learners’ scores on TOEIC, TOEFL, IELTS, or the English vocabulary size test (Nation and Beglar, 2007). In this respect, this corpus provides us with a large number of learner writing samples for analysis across relatively standard and widely accepted proficiency levels.

Secondly, all the writing essays, along with authors’ relevant metadata (e.g., age, gender, English type, and English level), can be freely accessed at ICNALE homepage ().^1^ Based on the metadata, subcorpora could be further extracted to accomplish our research objectives. In the present study, we chose to extract Chinese EFL learner data at A2_0, B1_1, and B1_2 CEFR proficiency levels since the sample size of B2_0 level in ICNALE is too limited to generate a robust result.

Thirdly, the ICNALE rigidly controls the prompts and tasks. The time for writing an essay and the length of an essay are controlled. In addition, topics are the same for all learners, who are required to express their opinions on two statements: (a) It is important for college students to have a part-time job; and (b) Smoking should be completely banned at all the restaurants in the country. These careful controls in the data collection process make the ICNALE a highly reliable database.

It should be noted that we combined the two written texts produced by the same learner into a new text since the calculation of Kolmogorov complexity is affected by the length of running texts (Ehret and Szmrecsanyi, 2016). In addition, these texts share similar task complexity features (e.g., production mode, argumentative writing genre, no contextual support, less planning time, and time pressured). In light of these similarities, we hold that there should be no difference between the two samples based on the Trade-off Hypothesis and Cognition Hypothesis (Foster and Skehan, 1996; Robinson, 2001). This step produced 387 texts, which were then used as the data. The statistical overview of the final data is shown in Table 1.

**TABLE 1:** Descriptive statistics of the corpus used in the study.

Proficiency	No. of texts	Words per text		Sentences per text	Total words
				
		Mean	SD		
A2_0	50	446.64	42.69	29.0	22,332
B1_1	232	470.71	53.26	29.7	109,204
B1_2	105	490.17	63.92	30.4	51,468
	**387**	**472.88**	**56.60**	**29.8**	**183,004**

### Metrics

#### Complexity metrics used in previous studies

We calculated a total of 17 complexity metrics used in previous studies, including traditional syntactic complexity from L2SCA (6), fine-grained clausal and phrasal complexity from TAASSC (9), and morphological complexity (2). It is necessary to note that the present study only considered syntactic and morphological complexity since no previous studies have proposed a metric, as our study did, to assess a text’s overall complexity.

As shown in Table 2, following Ouyang et al. (2022), we used six out of the fourteen traditional syntactic complexity metrics from L2SCA (Lu, 2010). Specifically, all three metrics addressing the length of production unit (i.e., MLS, MLC, and MLT) were chosen because they target distinct grammatical levels. In addition, we selected one metric in each of the other three dimensions (i.e., the amount of subordination, the amount of coordination, and the degree of phrasal sophistication), considering that some of the metrics are redundant (Norris and Ortega, 2009).

**TABLE 2:** The six traditional complexity metrics from L2SCA.

Metrics	Codes	Definitions
**Length of production unit**		
Mean length of clause	MLC	Number of words per clause
Mean length of sentence	MLS	Number of words per sentence
Mean length of T-unit	MLT	Number of words per T-units
**Amount of subordination**		
Dependent clauses per clause	DC/C	Number of dependent clauses per clause
**Amount of coordination**		
Coordinate phrases per clause	CP/C	Number of coordinate phrases per clause
**Degree of phrasal sophistication**		
Complex nominal per clause	CN/C	Number of complex nominals per clause

We also adopted nine fine-grained syntactic complexity metrics, including seven fine-grained phrasal metrics and two fine-grained clausal metrics (for details in Table 3), which have been proved effective in predicting learners’ writing quality in Kyle and Crossley (2018) and Zhang and Lu (2022). Note that both the traditional and the fine-grained syntactic complexity metrics were calculated by TAASSC (version 1.3.8; Kyle, 2016).

**TABLE 3:** The nine fine-grained complexity metrics from TAASSC.

Metrics	Codes	Definitions
Phrasal complexity		
Dependents per object of the preposition	av_pobj_deps	Number of dependents per object of the preposition
Prepositions per object of the preposition	prep_pobj_deps_struct	Number of prepositions per object of the preposition
Adjectival modifiers per object of the preposition	amod_pobj_deps_struct	Number of adjectival modifiers per object of the preposition
Dependents per direct object	av_dobj_deps	Average number of dependents per direct object
Dependents per direct object (standard deviation)	dobj_stdev	Standard deviation of the number of dependents per direct object
Dependents per nominal subject (standard deviation)	nsubj_stdev	Standard deviation of the number of dependents per nominal subject
Adjectival modifiers per nominal subject	amod_nsubj_deps_struct	Number of adjectival modifiers per nominal subject
Clausal complexity		
Nominal subjects per clause	nsubj_per_cl	Number of nominal subjects per clause
Adverbial modifiers per clause	advmod_per_cl	Number of adverbial modifiers per clause

Concerning morphological complexity, we adopted MCI (Brezina and Pallotti, 2019) and TTR. To be specific, MCI measures the inflectional diversity for a given word class (usually noun and verb class) within the text. For instance, the morphological complexity of a text that comprises *take*, *takes*, *taking* is considered to be greater than that of a text containing *taking*, *taking*, *taking* (or *making*, *thinking*, *writing*). MCI can be calculated using the Morpho complexity tool,^2^ which conducts two levels of analysis. First, linguistically, the tool identifies the word class of each word along with its specific inflectional form (exponence) in the text. Then, mathematically, the tool randomly samples all the exponences in a text and calculates the mean value of various exponences within and across these samples. In the present study, the calculation of MCI is based on 10-verb samples and includes both within and across sample diversity.

We computed TTR using TAALED 1.4.1 (Kyle and Crossley, 2015). TTR, a classic lexical diversity metric, was also adopted since Kettunen (2014) and Ehret (2021) proposed that TTR positively correlated with morphological complexity.

#### Calculation of the Kolmogorov complexity

According to Li et al. (2004) and Juola (2008), the Kolmogorov complexity of a text could be assessed by the length of the shortest description to reproduce it. In addition, Kolmogorov complexity could be approximated by some modern file compression programs such as gzip (Ziv and Lempel, 1977; Li et al., 2004). Specifically, texts that can be compressed more efficiently are regarded as linguistically comparatively simple, while texts that are less compressible are considered comparably more complex (Ehret and Szmrecsanyi, 2019).

String A, B, C, and D are shown below to illustrate how the algorithm of Kolmogorov complexity works. Although String A and B contain the same number of characters, i.e., 10 characters, String A can be compressed as 5 times *cd*, containing 4 characters, whereas String B cannot be compressed as it lacks any recurring pattern. As per Kolmogorov complexity, then, String A appears to be less complex than String B. Similarly, compared with String D, String C can be described more efficiently since the pattern *there are great* occurs twice.

A. cdcdcdcdcd (10 characters) – 5 × cd (4 characters).
B. ncslv73pds (10 characters) – ncslv73pds (10 characters).
C. There are great holes and there are great caverns in an icy mountain
(56 characters; adapted from sentence D to facilitate understanding).
D. There are great holes and caverns which are made when the ice bursts
(56 characters; extracted from *Royal Society Corpus 6.0 Open*).

Inspired by Juola (2008), Ehret (2017: 43–82) introduced a compression technique to facilitate the application of Kolmogorov complexity to linguistic data. In the present study, we replicated the pioneering work of Ehret (2017: 43–82) and Ehret and Szmrecsanyi (2019: 27–30) in terms of the compression technique and the procedures for Kolmogorov complexity calculation. Specifically, gzip (GNU zip, Version 1.11)^3^ was used to approximate the Kolmogorov complexity of each text in terms of the overall, syntactic, and morphological level. In addition, the original algorithm of the compression technique is available on GitHub: *https://github.com/katehret/measuring-language-complexity.*

(1)Overall complexity

A text’s overall complexity is consistent with Miestamo et al.’s (2008) notion of global complexity, which consists of the complexity of all levels of a language (e.g., morphology and syntax), thus addressing the global structural complexity of a text as a whole.

To calculate the overall complexity, we first measured each text’s file size before and after compression. Following that, a linear regression analysis was conducted by taking uncompressed file size as the independent variable and compressed file size as the dependent variable, thus eliminating the correlation between them. This step generates the adjusted overall complexity scores (i.e., regression residuals) of sample texts: higher scores suggest higher overall linguistic complexity, while lower scores indicate lower complexity.

(2) Morphological complexity

As Juola (2008) observed, a text’s morphological and syntactic complexity can be indirectly assessed by distorting text files before compression. Thus, to measure the morphological complexity, we first randomly deleted 10% of the characters before applying compression. This proportion (i.e., 10%) is commonly adopted in previous literature (Juola, 1998; Sadeniemi et al., 2008; Ehret and Taboada, 2021). Then we compressed the distorted texts to determine how well or badly the compression technique handles the distortion. Formula 1 shows the algorithm of morphological complexity.

Formula 1

Morphological complexity score = $\frac{m}{c}$

In Formula 1, *m* represents the compressed file size after morphological distortion, and *c* indicates the original compressed file size. As morphologically complex texts typically contain more word forms, they will be less affected by distortion compared to morphologically simple texts. Therefore, comparatively bad compression ratios after morphological distortion indicate low morphological complexity, and vice versa.

(3) Syntactic complexity

To calculate syntactic complexity, we randomly deleted 10% of all word tokens in each text. Then we compressed the distorted texts and obtained the syntactic complexity scores of given texts according to Formula 2, in which *s* represents the compressed file size after syntactic distortion, and *c* is the file size before distortion.

Formula 2

Syntactic complexity score = $-\frac{s}{c}$

It is worth noting that syntactic complexity in the present study is measured by word order rigidity (Bakker, 1998): rigid word order signifies syntactically complex texts, whereas free word order indicates syntactically simple texts. Syntactic distortion, then, disrupts word order regularities, resulting in random noises. Syntactically complex texts are greatly affected, and their compression efficiency is compromised; syntactically simple texts, in contrast, are less affected due to a lack of syntactic interdependencies that could be compromised. As a result, comparatively bad compression ratios after syntactic distortion indicate high Kolmogorov syntactic complexity. This seems to be counterintuitive because free word order with lower predictability ought to be more complex than rigid word order. However, we should keep in mind that, in the present study, Kolmogorov syntactic complexity is calculated indirectly since we assess to what extent distortion will influence the predictability of a text. If the predictability of a text decreases after distortion, the text is regarded as syntactically complex. As a result, rigid word order is considered as Kolmogorov complex from a technical perspective (Ehret and Szmrecsanyi, 2019: 28).

### Data processing

The procedures of data processing are described in this section. Note that all the procedures have been achieved by homemade scripts in R, a programming language for data processing and statistical analysis.

#### Data collection

We extracted all the 774 written samples produced by EFL learners from China across three proficiency levels (i.e., A2_0, B1_1, and B1_2). Then we integrated the two texts written by the same learner into a new text. This step produced 387 texts, which were then used as the data.

#### Data cleaning

We cleaned the data by lowercasing all the running texts and removing non-alphabetical characters (e.g., numbers, UTF-8 characters, and corpus markups) and punctuations (e.g., dashes, commas, and hyphens). We did this because punctuations and non-alphabetical characters would compromise the compressibility of texts and thus increase their complexity. Notably, we retained the full stops and replaced other end-of-sentence markings (e.g., question marks, exclamation marks, and semicolons) with full stops. This is because full stops serving as the end markers of sentences are used to determine the linguistic units of random sampling in Kolmogorov complexity calculation. Furthermore, we have also manually checked all the possible mistakes due to the deletion of numbers and punctuations.

#### Kolmogorov complexity calculation

To generate a statistically robust result, we repeated the distortion and compression process for each text 500 times. In each iteration, we employed random sampling, that is, randomly selected five sentences per text. We did this because random sampling keeps sample size constant, thus ensuring the comparability of linguistic metrics among texts of different sizes.

To measure the overall Kolmogorov complexity, we calculated the mean file sizes before and after compression across all iterations. Subsequently, a linear regression was performed, and the adjusted overall complexity scores for each text were calculated. For the morphological and syntactic complexity, we firstly calculated their scores for each text file in each iteration. Then, the average morphological and syntactic complexity scores for each text were computed across all iterations, respectively.

### Statistical analyses

The Shapiro–Wilk tests and Q-Q plots showed that almost all complexity metrics were not normally distributed except for MCI and TTR. The one-way ANOVA test was used to determine whether there was a significant difference in the MCI and TTR across the three proficiency levels. As for other complexity metrics, the Kruskal–Wallis tests were performed to determine whether the differences were significant in these metrics across the three levels. The Holm’s *post-hoc* test was then used to examine whether a significant difference could be traced between every two adjacent levels.

To determine the possible error resulting from the internal heterogeneity of different L2 levels, we performed two independent-samples Mann–Whitney *U* tests on three Kolmogorov complexity metrics at two pseudo-levels of each L2 level. Specifically, each L2 level (i.e., beginner level, lower-intermediate level, and upper-intermediate level) is divided equally into two pseudo-levels: Group 1 and Group 2. Furthermore, a visual inspection of distributions and the Shapiro–Wilk normality tests suggested that some Kolmogorov complexity metrics did not conform to the normal distribution. For the sake of consistency, Mann–Whitney *U* tests were adopted on all three Kolmogorov complexity metrics.

In addition, the Pearson product-moment correlation coefficient analysis was conducted to further assess the association between all the linguistic complexity metrics used in the present study.

## Results

In this section, we first presented the statistical results for linguistic complexity metrics at three levels (i.e., overall complexity, syntactic complexity, and morphological complexity) in predicting L2 proficiency. Secondly, we provided the results of Mann–Whitney *U* tests on three Kolmogorov complexity metrics at two pseudo-levels of each L2 level. Then, the results of correlation tests were reported to reveal the interrelation between linguistic complexity metrics. The descriptive statistics for all complexity metrics (i.e., mean and standard deviation per proficiency level) are available in Supplementary material.

### The relationship between overall complexity and L2 proficiency

**FIGURE 1:**
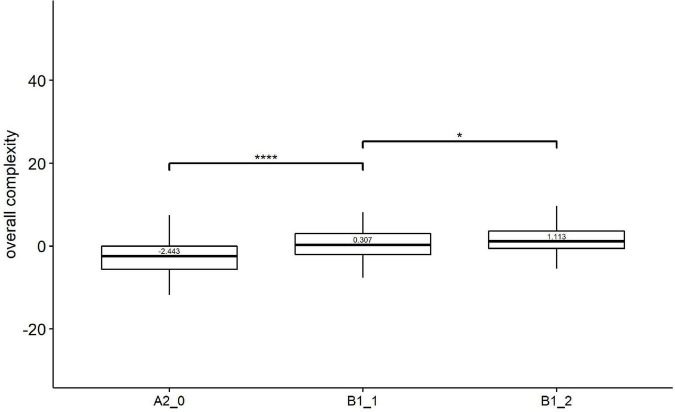
Paired comparisons across learner proficiency levels on Kolmogorov overall complexity. *p < 0.01; ****p < 0.0001.

As shown in Figure 1, the median score of Kolmogorov overall complexity increased with the development of L2 proficiency. Kruskal–Wallis showed a significant effect of learner proficiency on Kolmogorov overall complexity [*H*(2) = 35.50, *p* = 0.000, *eta2[H]* = 0.087]. Follow-up paired comparisons (Figure 1) revealed that there were significant differences between all two pairs of adjacent levels, i.e., beginner level (A2_0) vs. lower-intermediate level (B1_1), and lower-intermediate level (B1_1) vs. upper-intermediate level (B1_2).

### The relationship between syntactic complexity and L2 proficiency

**FIGURE 2:**
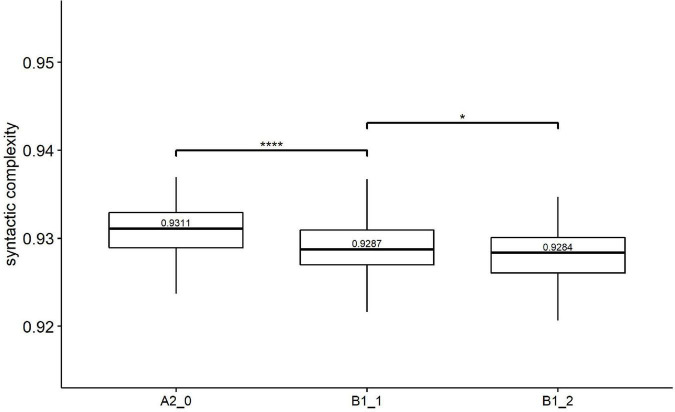
Paired comparisons across learner proficiency levels on Kolmogorov syntactic complexity. *p < 0.01; ****p < 0.0001.

**FIGURE 3:**
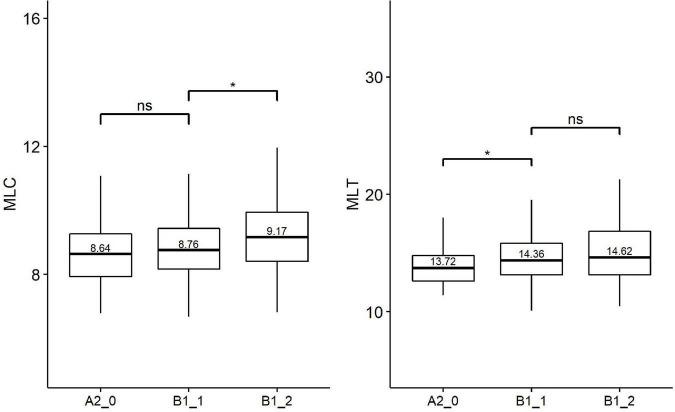
Paired comparisons across learner proficiency levels on traditional syntactic complexity. *p < 0.01; ns, no significant difference.

**FIGURE 4:**
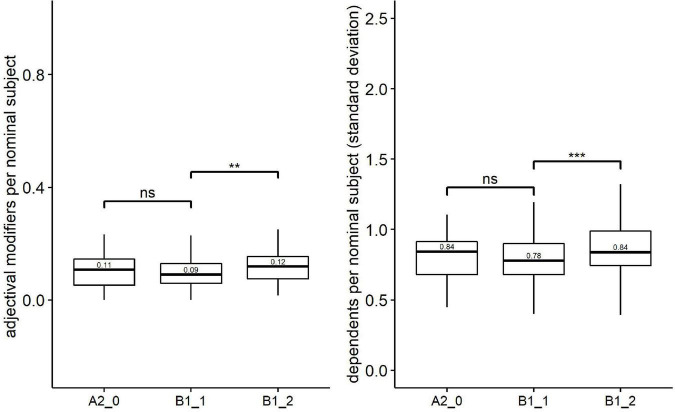
Paired comparisons across learner proficiency levels on fine-grained syntactic complexity. **p < 0.001; ***p < 0.0001; ns, no significant difference.

Table 4 provides the results of Kruskal-Wallis tests on three groups of syntactic complexity metrics (i.e., Kolmogorov syntactic complexity, traditional syntactic complexity, and fine-grained syntactic complexity). Based on Holm’s *post-hoc* tests, Figures 2–4 shows the significance level between any two adjacent levels as well as the median scores of each complexity metric.

**TABLE 4:** Differences in syntactic complexity metrics among A2_0, B1_1, and B1_2.

	Complexity metrics	*H*	Sig	*eta2[H]*
Kolmogorov complexity	Kolmogorov syntactic complexity	30.23	0.000****	0.074
Traditional syntactic complexity	CN_C	9.61	0.008*	0.020
	CP_C	6.51	0.039*	0.012
	DC_C	2.89	0.236	0.002
	MLC	10.60	0.005*	0.022
	MLS	4.91	0.086	0.008
	MLT	9.01	0.011*	0.018
Fine-grained syntactic complexity	advmod_per_cl	4.24	0.120	0.006
	amod_nsubj_deps_struct	8.73	0.013*	0.018
	amod_pobj_deps_struct	2.34	0.310	0.001
	av_dobj_deps	0.01	0.995	–0.005
	av_pobj_deps	2.03	0.362	0.000
	dobj_stdev	0.46	0.796	–0.004
	nsubj_per_cl	3.25	0.197	0.003
	nsubj_stdev	13.61	0.001**	0.030
	prep_pobj_deps_struct	2.68	0.261	0.002

*p < 0.05; **p < 0.005; ****p < 0.00005; a detailed description of traditional syntactic complexity metrics and fine-grained syntactic complexity metrics were provided in Tables 2, 3, respectively.

Regarding Kolmogorov complexity, Figure 2 shows that the median value of Kolmogorov syntactic complexity decreased with increasing proficiency levels. Kruskal–Wallis (see Table 4) showed significant effects of proficiency levels on Kolmogorov syntactic complexity [*H*(2) = 30.23, *p* = 0.000, *eta2[H]* = 0.074]. The subsequent Holm’s *post-hoc* tests (Figure 2) revealed significant differences between all two pairs of adjacent levels in Kolmogorov syntactic complexity, i.e., beginner level (A2_0) vs. lower-intermediate level (B1_1) and lower-intermediate level (B1_1) vs. upper-intermediate level (B1_2).

As for the traditional syntactic complexity, Kruskal–Wallis indicates significant effects of proficiency levels on four metrics: CN/C [*H*(2) = 9.61, *p* = 0.008, *eta2[H]* = 0.020], CP/C [*H*(2) = 6.51, *p* = 0.040, *eta2[H]* = 0.012], MLC [*H*(2) = 10.60, *p* = 0.005, *eta2[H]* = 0.022], MLT [*H*(2) = 9.01, *p* = 0.011, *eta2[H]* = 0.018]. Follow-up paired comparisons (Figure 3) showed that there were significant differences between only one pair of adjacent levels in the MLT (beginner level vs. lower-intermediate level) and the MLC (lower-intermediate level vs. upper-intermediate level). None of the traditional syntactic complexity metrics could differentiate all two pairs of adjacent levels.

For the fine-grained syntactic complexity, Kruskal–Wallis revealed significant effects of proficiency levels on two metrics: adjectival modifiers per nominal subject [*H*(2) = 8.73, *p* = 0.013, *eta2[H]* = 0.018] and dependents per nominal subject (standard deviation) [*H*(2) = 13.61, *p* = 0.001, *eta2[H]* = 0.030]. Follow-up paired comparisons (Figure 4) showed that both metrics could distinguish one pair of proficiency levels, i.e., lower-intermediate level (B1_1) vs. upper-intermediate level (B1_2).

### The relationship between morphological complexity and L2 proficiency

Kruskal–Wallis showed significant effects of proficiency levels on all three morphological complexity metrics: Kolmogorov morphological complexity [*H*(2) = 20.24, *p* = 0.000, *eta2[H]* = 0.050], MCI [*F*(2, 384) = 3.58, *p* = 0.029, *eta2* = 0.018], and TTR [*F*(2, 384) = 5.50, *p* = 0.005, *eta2* = 0.028]. Among these metrics, Kolmogorov morphological complexity had the biggest effect size with a small magnitude (< 0.06), nevertheless. Then, as indicated by the follow-up paired comparisons, both TTR and Kolmogorov morphological complexity was found to be significantly different in one adjacent pair of proficiency levels: beginner level (A2_0) vs. lower-intermediate level (B1_1). For MCI, no significant difference was detected between any pair of adjacent levels.

### Kolmogorov complexity differences between two pseudo-level groups

To figure out the possible error resulting from the internal heterogeneity of different L2 levels, Mann–Whitney *U* tests were adopted on three Kolmogorov complexity metrics at two pseudo-level groups of each L2 level. Table 5 shows that there was no statistically significant difference (*p* > 0.05) in the values of all three Kolmogorov complexity metrics (i.e., Kolmogorov overall complexity, Kolmogorov syntactic complexity, and Kolmogorov morphological complexity) between the pseudo-level Group 1 and Group 2 in each L2 level. These results indicated that Kolmogorov complexity at all three sublevels is relatively consistent within any L2 level, thus further corroborating the validity of differences found between any adjacent proficiency levels.

**TABLE 5:** Mann–Whitney *U* results on each proficiency level between two pseudo-level groups.

Level	Metrics	Mean Rank		U	W	Z	*p*
						
		Group 1	Group 2				
A2_0	Overall	26.16	24.84	296.000	621.000	–0.320	0.749
	Morphological	27.48	23.52	263.000	588.000	–0.960	0.337
	Syntactic	25.08	25.92	302.000	627.000	–0.204	0.839
B1_1	Overall	117.03	115.97	6667.000	13453.000	–0.119	0.905
	Morphological	119.28	113.72	6406.000	13192.000	–0.630	0.529
	Syntactic	113.59	119.41	6391.000	13177.000	–0.659	0.510
B1_2	Overall	47.87	58.04	1111.000	2489.000	–1.711	0.087
	Morphological	50.75	55.21	1261.000	2639.000	–0.750	0.453
	Syntactic	56.4	49.66	1201.000	2632.000	–1.134	0.257

### Correlation between linguistic complexity metrics

**FIGURE 5:**
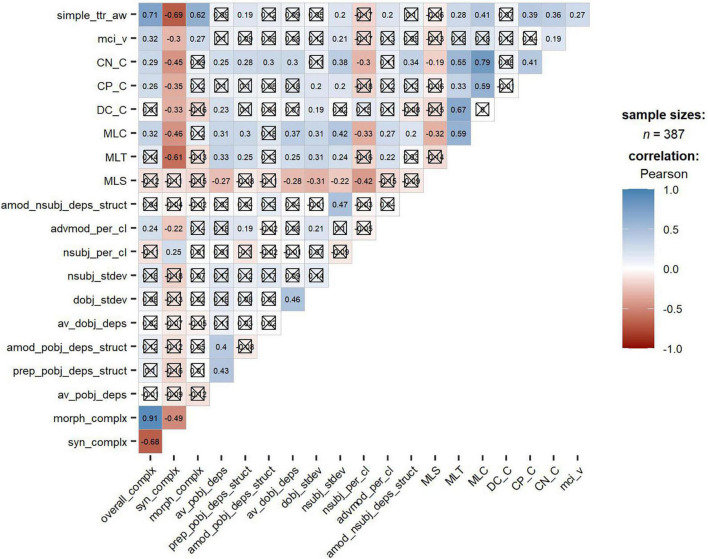
Correlation among linguistic complexity metrics. The square cross indicates the associated *p*-values are less than 0.05, so that no significant association is detected between the two metrics; As reported by Wolfe-Quintero et al. (1998), correlations can be marked as strong (*r* ≥ 0.65), moderate (0.45 ≤ *r*< 0.65), and weak (0.25 ≤ *r*< 0.45).

Figure 5 exhibits the correlation among all the 20 linguistic complexity metrics at three levels (i.e., overall complexity, syntactic complexity, and morphological complexity). The correlation coefficient for each pair of complexity metrics was provided in each cell. Note that the deeper the cell’s color, the stronger the correlation between the two metrics.

Results revealed some important points worth noting. First, three Kolmogorov complexity metrics were observed to closely relate to each other. Specifically, there was a very strong positive relationship between Kolmogorov overall complexity and Kolmogorov morphological complexity (*r* = 0.91), while a relatively strong negative relationship was detected between Kolmogorov overall complexity and Kolmogorov syntactic complexity (*r* = –0.68). In addition, there was a moderate negative relationship between Kolmogorov morphological complexity and Kolmogorov syntactic complexity (*r* = –0.49).

Second, mixed results were revealed between Kolmogorov complexity and other complexity metrics at different levels. For instance, at the overall level, Kolmogorov overall complexity was strongly correlated with TTR (*r* = 0.71). Regarding syntactic complexity, a moderate negative relationship was found between Kolmogorov syntactic complexity and traditional syntactic complexity metrics such as MLC (*r* = –0.46) and MLT (*r* = –0.61). In contrast, Kolmogorov syntactic complexity was not significantly correlated with seven fine-grained phrasal complexity metrics and weakly correlated with two fine-grained clausal complexity metrics (i.e., adverbial modifiers per clause, *r* = –0.22; nominal subjects per clause, *r* = 0.25). As per morphological complexity, the Kolmogorov morphological complexity displayed a much stronger positive relationship with TTR (*r* = 0.62) than MCI (*r* = 0.27).

## Discussion

### Effects of complexity metrics in differentiating L2 proficiency

Generally speaking, our results showed that the Kolmogorov overall and syntactic complexity metrics are capable of significantly differentiating all pairs of adjacent levels, making them the strongest predictors to discriminate the proficiency of beginner, lower-intermediate, and upper-intermediate learners. By contrast, no other complexity metrics could significantly distinguish all pairs of adjacent levels. In addition, our results showed no significant difference in all three Kolmogorov complexity metrics within the same-level data, which further demonstrated their reliability in differentiating L2 proficiency.

Taking a closer look at the results per complexity level, we found that for overall complexity, the Kolmogorov overall complexity is a good candidate as an index of proficiency. Specifically, the Kolmogorov overall complexity increased consistently from the beginner level (A2_0) to the upper-intermediate level (B1_2), significantly discriminating each pair of proficiency levels. These results may indicate that EFL learners tend to produce overall complex texts with increasing proficiency levels.

Regarding syntactic complexity, our study confirmed that the Kolmogorov syntactic complexity could significantly distinguish any pair of proficiency levels. With the increase of learner proficiency from the beginner level (A2_0) to the upper-intermediate level (B1_2), the Kolmogorov syntactic complexity declined consistently, which reveals that learners with higher proficiency tend to produce texts containing more various word order patterns. This result supported Ehret and Szmrecsanyi’s (2019) findings that the writing of EFL learners is characterized by decreased syntactic complexity (defined here as less rigid word order) with the increase in L2 instructional exposure, which may be interpreted by the fact that as learners receive more instruction and improve their proficiency levels, they use more varied word order patterns.

Among the traditional syntactic complexity metrics, two length of production unit metrics (i.e., MLC and MLT), which significantly distinguish one pair of adjacent levels, serve as the better indicators of learner proficiency as compared with other syntactic complexity metrics from L2SCA. This finding agrees with previous studies (Wolfe-Quintero et al., 1998; Lu, 2011; Bulté and Housen, 2014; Gyllstad et al., 2014). For instance, Lu (2011) found that MLC performed best among the 14 traditional syntactic complexity metrics in discriminating different proficiency levels. Furthermore, we found that MLC could not distinguish beginner level (A2_0) from lower-intermediate (B1_1), which is likely because the MLC is primarily determined by the use of phrases in clauses (Bulté and Housen, 2012; Alexopoulou et al., 2017). As illustrated in our findings concerning the fined-grained syntactic complexity metrics, no sizeable differences have been detected between beginner level (A2_0) and lower-intermediate level (B1_1) in all seven fined-grained phrasal complexity metrics explored in the present study.

In terms of the fine-grained syntactic metrics, only two fine-grained phrasal syntactic complexity metrics [i.e., the median scores of the adjectival modifiers per nominal subject and the dependents per nominal subject (standard deviation)] could significantly distinguish a pair of adjacent proficiency levels (lower-intermediate level vs. upper-intermediate level). However, no significant difference was found in fine-grained clausal complexity metrics across proficiency levels. These findings are consistent with Kyle and Crossley (2018), who argued that metrics of phrasal complexity were more appropriate for measuring L2 writing proficiency compared with metrics of clausal complexity. In addition, Biber et al. (2011) proposed that phrasal complexity was a distinctive feature of writing, whereas clausal complexity was a distinctive feature of conversations.

Concerning morphological complexity, our results showed that both TTR and the Kolmogorov morphological complexity increased consistently as proficiency improved and could distinguish between beginner level (A2_0) and lower-intermediate level (B1_1). These results are in line with Ehret and Szmrecsanyi (2019), who argued that writings produced by more proficient learners exhibit more word forms and/or derivational forms than those of less proficient learners.

However, both TTR and the Kolmogorov morphological complexity could not distinguish between lower-intermediate level (B1_1) and higher-intermediate level (B1_2). This result may be explained by the combined effects of learner proficiency and the prompts controlled in the present study. Specifically, B1_1 and B1_2 are two subcategories of the intermediate level, thus making their usage of word forms highly similar. In addition, the controlled topics (i.e., part-time jobs for college students and non-smoking at restaurants) and limited length (200–300 words) for sample writing may also constrain the various usage of word forms in B1_1 and B1_2 levels.

It is worth noting that no significant difference was detected between any adjacent levels in MCI. Such a result may be attributed to L2 learners’ proficiency and the particular language under discussion. Specifically, for the language with limited inflectional resources (e.g., English), learners will soon reach a threshold proficiency level, after which inflectional diversity remains constant (Brezina and Pallotti, 2019).

### Correlation between linguistic complexity metrics

As inspired by Lu (2011), investigating the correlations between complexity metrics can help us better understand why some of them display similar patterns in predicting learners’ proficiency levels (e.g., increase/decrease as the proficiency goes up). Moreover, these correlations can reveal the metrics targeting different linguistic aspects, thus contributing to identifying metrics that should be selected together for distinguishing different proficiency levels.

Our results showed that, firstly, regarding the three Kolmogorov complexity metrics (i.e., overall, syntactic, and morphological complexity), there was a strong positive correlation between Kolmogorov morphological complexity and overall complexity. Such a result, as explained by Ehret and Szmrecsanyi (2016), is possibly due to the similar nature of the algorithm, which detects surface structure irregularities or redundancy of the running texts.

Furthermore, a moderate negative correlation was detected between Kolmogorov morphological complexity and syntactic complexity, which may reveal a complementary relationship in L2 writing. In other words, if the morphology of a writing is complex enough, syntax, as compensation, might be simplified for the efficiency of communication. This result is consistent with those observed in earlier studies (Ehret and Szmrecsanyi, 2016, 2019; Ehret and Taboada, 2021; Sun et al., 2021). For example, based on the International Corpus of Learner English (ICLE), Ehret and Szmrecsanyi (2019) have noticed a statistically significant negative correlation between syntactic and morphological complexity in essays written by EFL learners with different L2 instructional exposure. Such a trade-off has been observed not only in L2 writing but also in other types of texts such as literary works (Ehret and Szmrecsanyi, 2016), newspaper texts (Ehret and Taboada, 2021), and scientific writing (Sun et al., 2021).

Second, there are some interesting findings concerning the relationship between Kolmogorov complexity and other complexity metrics at different levels. At the overall level, Kolmogorov overall complexity displayed a strong positive correlation with TTR suggesting that Kolmogorov overall complexity overlaps with metrics addressing lexical diversity, which accords with Ehret (2021). This result may be attributed to the fact that in linguistic terms, Kolmogorov complexity is a metric of structural redundancy and therefore inherently associated with any structural (ir)regularities in the text, whether at morphological or lexical levels.

At the syntactic level, our Kolmogorov syntactic complexity was not significantly correlated with seven fine-grained phrasal complexity metrics and weakly correlated with two fine-grained clausal complexity metrics. These results may result from the fact that Kolmogorov syntactic complexity and fine-grained complexity metrics explain linguistically different aspects. Specifically, fine-grained complexity metrics are feature-specific, as they target particular phrasal (e.g., determiners, adjective modifiers, and nouns as modifiers) and clausal structures (e.g., adjective complement, adverb modifier, and clausal complement) in writings. On the contrary, Kolmogorov syntactic complexity is not feature-specific but global, as it takes the entire structural complexity of texts into consideration. It is a measure of word order flexibility and indicates to what extent word order in a text is flexible or rigid. Therefore, there is no or just a weak correlation between Kolmogorov syntactic complexity and fine-grained syntactic complexity metrics. In addition, as Mendelsohn (1983) reported that a weak correlation between two metrics suggests that they capture different aspects of development, thus both should be considered in describing learners’ proficiency levels. As a result, it may be advisable to combine Kolmogorov syntactic complexity with fine-grained clausal or phrasal complexity to discriminate L2 proficiency.

Regarding morphological complexity, the Kolmogorov morphological complexity displayed a strong positive relationship with TTR, indicating that both metrics are capable of capturing differences related to various word forms. Such a relationship has also been addressed in the study of Ehret and Szmrecsanyi (2016) and Ehret (2021). In addition, MCI is found to have a weaker correlation with Kolmogorov morphological complexity than TTR. A possible explanation for this may lie in that MCI only measures the inflectional diversity for a given word class (the verb class in our study) within the text, while Kolmogorov morphological complexity is about all word form variations which include and is not limited to both inflectional and derivational morphology (Ehret, 2021).

## Conclusion

The present study applied a novel Kolmogorov complexity derived from information theory and examined its validity in differentiating EFL learners’ proficiency levels by comparing it with other complexity metrics demonstrated to be effective in previous studies. Specifically, based on 774 argumentative writings produced by Chinese EFL learners, we have investigated to what extent traditional syntactic and morphological complexity metrics, fine-grained syntactic complexity, and Kolmogorov complexity metrics (i.e., Kolmogorov overall, syntactic, and morphological complexity) can differentiate the proficiency of L2 beginner, lower-intermediate, and higher-intermediate learners. In addition, we have also explored the correlations between all the complexity metrics at different levels to determine which metrics should be selected in predicting proficiency levels.

For the first question, it turned out that at the overall level, Kolmogorov overall complexity was a good indicator of L2 learners’ proficiency since significant differences were observed between any two adjacent levels (i.e., beginner vs. lower-intermediate levels and lower-intermediate vs. upper-intermediate levels). Concerning syntactic complexity, Kolmogorov syntactic complexity was the only metric capable of differentiating any pair of adjacent proficiency levels. In addition, one length-based traditional syntactic complexity metric (i.e., MLC) and two fine-grained phrasal complexity metrics [i.e., adjectival modifiers per nominal subject and the dependents per nominal subject (standard deviation)] could distinguish lower-intermediate and upper-intermediate levels, while MLT could distinguish beginner and lower-intermediate levels. At the morphological level, TTR and Kolmogorov morphological complexity perform best in discriminating L2 proficiency, although both could only separate one adjacent pair of levels (i.e., beginner level vs. lower-intermediate level).

For the second question, we observed a moderate negative correlation between Kolmogorov morphological complexity and Kolmogorov syntactic complexity, revealing a trade-off between morphology and syntax in L2 writing. In addition, our Kolmogorov morphological complexity was found to be more strongly correlated with TTR than MCI as it captures variations in both inflectional and derivational forms, while MCI used in the present study merely targets at inflectional diversity of verbs. Furthermore, Kolmogorov syntactic complexity did not demonstrate any significant correlation with any of the seven fine-grained phrasal sophistication metrics, indicating that they may detect distinctive linguistic features, with Kolmogorov syntactic complexity assessing word order flexibility while fine-grained metrics dealing with particular phrasal structures.

The findings of this study have some implications for assessing large-scale writing data. Specifically, compared with metrics used in previous studies, Kolmogorov complexity is more global and comprehensive because it is capable of gauging three dimensions (i.e., overall, syntactic and morphological complexity) of L2 proficiency simultaneously. Therefore, Kolmogorov complexity is well suited for capturing the complex multi-dimensional nature of L2 complexity (Ehret and Szmrecsanyi, 2016). Moreover, Kolmogorov complexity, as a holistic and quantitative approach to text complexity, is both more convenient to operate and arguably more objective than, for example, subjective complexity ratings of learner texts by expert evaluators.

Some limitations of this study should be recognized. First, it is worth noting that Kolmogorov complexity metrics are insufficient in detecting the changes of specific linguistic features compared with fine-grained syntactic complexity metrics. However, considering the significant effect of Kolmogorov syntactic complexity metrics in differentiating learner proficiency, we propose that Kolmogorov complexity metrics may complement the fine-grained ones to better depict the development of linguistic complexity across proficiency levels. Second, we have only selected argumentative writings produced by Chinese EFL learners as the data. Future studies may consider diverse first language backgrounds and genres to further evaluate the validity and reliability of this methodology. In addition, only three proficiency levels were covered in the present study, and future research could involve more levels to validate the reliability of Kolmogorov complexity as well as to gain a fuller picture of the relationship between linguistic complexity and L2 levels.

## Data availability statement

Publicly available datasets were analyzed in this study. This data can be found here: http://language.sakura.ne.jp/icnale/.

## Author contributions

HW performed the material preparation and data collection. GW carried out data processing. GW and HW wrote the first draft of the manuscript. All authors especially LW commented on previous versions of the manuscript, contributed to the study conception and design, read, and approved the final manuscript.

## Footnotes

1
http://language.sakura.ne.jp/icnale/

2
http://corpora.lancs.ac.uk/vocab/analyse_morph.php

3
https://ftp.gnu.org/gnu/gzip/
