# Supplementary material for: Kolmogorov complexity metrics in assessing L2 proficiency: An information-theoretic approach
Source: Front Psychol. 2022 Oct 6;13:1024147. doi: 10.3389/fpsyg.2022.1024147 (PMC9583672; doi:10.3389/fpsyg.2022.1024147)
Supplement: Supplementary file 1 [file Table_1.DOCX]

The mean and standard deviation (in parentheses) of complexity metrics.

|  | **Proficiency level** | | |
| --- | --- | --- | --- |
|  | A2_0 | B1_1 | B1_2 |
| **Kolmogorov complexity** |  |  |  |
| Morphological complexity | -0.964 (0.01) | -0.959 (0.01) | -0.958 (0.01) |
| Overall complexity | -3.60 (5.42) | 0.19 (4.70) | 1.30 (4.25) |
| Syntactic complexity | 0.931 (0.01) | 0.929 (0.01) | 0.928 (0.01) |
| **Fine-grained syntactic complexity** |  |  |  |
| adverbial modifiers per clause | 0.21 (0.07) | 0.23 (0.07) | 0.24 (0.07) |
| adjectival modifiers per nominal subject | 0.10 (0.06) | 0.10 (0.05) | 0.12 (0.06) |
| adjectival modifiers per object of the preposition | 0.16 (0.09) | 0.17 (0.07) | 0.17 (0.06) |
| dependents per direct object | 1.32 (0.30) | 1.32 (0.21) | 1.34 (0.22) |
| dependents per object of the preposition | 1.03 (0.22) | 1.02 (0.17) | 1.03 (0.15) |
| dependents per direct object (standard deviation) | 0.98 (0.21) | 0.99 (0.14) | 1.00 (0.13) |
| nominal subjects per clause | 0.65 (0.12) | 0.67 (0.08) | 0.65 (0.08) |
| dependents per nominal subject (standard deviation) | 0.80 (0.18) | 0.78 (0.16) | 0.86 (0.18) |
| prepositions per object of the preposition | 0.10 (0.05) | 0.11 (0.05) | 0.10 (0.05) |
| **Traditional syntactic complexity** |  |  |  |
| CN_C | 0.96 (0.23) | 1.02 (0.19) | 1.08 (0.23) |
| CP_C | 0.13 (0.06) | 0.15 (0.07) | 0.17 (0.10) |
| DC_C | 0.35 (0.09) | 0.38 (0.07) | 0.37 (0.08) |
| MLC | 8.57 (1.63) | 8.88 (1.02) | 9.27 (1.25) |
| MLS | 23.82 (59.12) | 16.31 (3.35) | 16.63 (3.32) |
| MLT | 13.83 (2.97) | 14.86 (2.63) | 15.18 (2.64) |
| T_S | 1.07 (0.18) | 1.10 (0.09) | 1.10 (0.13) |
| **Traditional morphological complexity** |  |  |  |
| MCI | 3.99 (0.68) | 4.22 (0.72) | 4.32 (0.72) |
| TTR | 0.40 (0.042) | 0.42 (0.04) | 0.43 (0.04) |
